# The clinical trials on gamma globulin for polio: victims of marketing success

**DOI:** 10.1503/cmaj.170232

**Published:** 2017-07-24

**Authors:** Stephen E. Mawdsley

**Affiliations:** Centre for the Social History of Health and Healthcare, University of Strathclyde, Glasgow, Scotland

During the early 1950s, American researchers enrolled more than 55 000 healthy children in a medical experiment to assess whether the blood fraction, gamma globulin, could be used as a means to prevent paralytic polio.^1^–^6^ Because a large, double-blind, placebo-controlled study had never been attempted on an open population before, publicity became an inextricable part of the design.^7^ Although marketing the experiment was successful and clinic attendance surpassed researchers’ expectations, the enthusiasm of parents and health professionals led to unforeseen consequences. Researchers struggled to maintain control of the experiment and subsequently suppressed the extent of the problems and did not acknowledge the implications for medical science. The successes and setbacks of this study later influenced the Salk vaccine trials in 1954, while also raising important questions about the conduct of one of the first “gold standard” clinical trials.

Before a safe and effective polio vaccine was licensed in April 1955, Americans faced the constant threat of polio outbreaks. Caused by an oral-fecal virus, a polio infection could lead to paralysis of the limbs and respiratory muscles or, in extreme cases, death. Although anyone could contract the disease, children seemed the most susceptible, inspiring the term “infantile paralysis.”^8^,^9^ Desperate for a means to prevent polio disability, the National Foundation for Infantile Paralysis (now known as the March of Dimes) backed University of Pittsburgh researcher Dr. William McD. Hammon to undertake a controlled study with gamma globulin. The blood fraction, rich in antibodies, was already used for the prevention of measles and hepatitis and was therefore considered safe. Hammon hoped that by randomly injecting 4 mL to 11 mL of either placebo solution or gamma globulin into the gluteus maximus of children and comparing the two groups, the value of the blood fraction for polio could be established.

**Figure:**
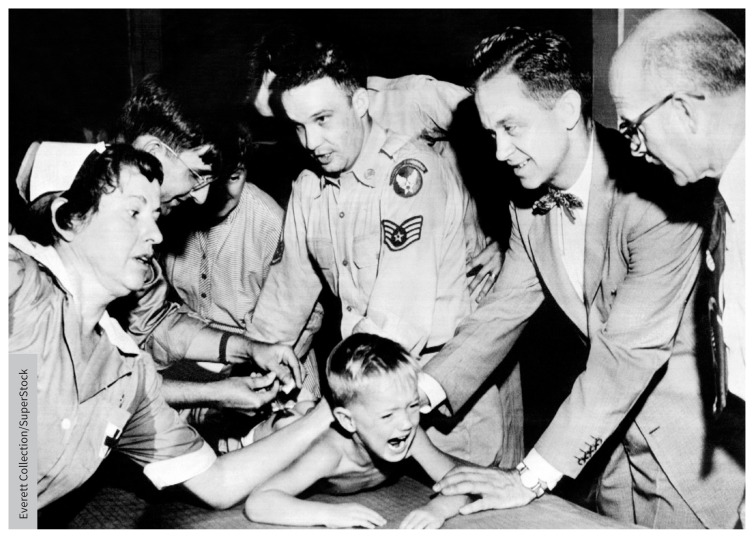
Doctors and nurses restrain Ernest Booth, one of 30 000 children to receive a gamma globulin inoculation during the polio epidemic in Houston, Texas, in 1952.

Image courtesy of Everett Collection/SuperStock

Hammon and foundation officials believed that a publicity program was necessary to encourage parents to volunteer their healthy children. The foundation drew on its vast marketing expertise and connections to develop a sophisticated campaign for Hammon’s experiment. Unlike the annual March of Dimes polio fundraising drive, which benefited from weeks of advance publicity, promotion of the gamma globulin trial would be limited to days, because it was difficult to predict an epidemic; and once identified, the epidemic would not last long. Moreover, parents would need to be educated to appreciate the value of controlled trial methodology, in which only half the cohort would receive the potentially protective substance. Hammon believed that any protection offered by gamma globulin would last only a few weeks, so it had to be administered at the early stages of an epidemic, when passive immunization could offer the most protection. Because parents could decide whether or when to volunteer their children, the faster the maximum cohort was achieved, the greater the opportunity to assess gamma globulin. The field team needed to react quickly and be prepared to inject thousands of children in temporary clinics. By anticipating parental anxieties in publicity materials, foundation officials hoped to provide Hammon with steady enrolment and protocol compliance.

When a high-incidence polio epidemic erupted in Utah in 1951, Hammon and foundation officials launched their experiment. They moved supplies to a staging site in Provo and instigated a publicity program to build local support. Because of state medical licensing laws, only Utah doctors could administer the test sera, making it necessary for Hammon to direct his initial publicity efforts to physicians. To Hammon’s astonishment, local doctors made the experiment their own, seeing participation as a professional duty and a form of public health activism. They not only agreed to administer the injections, but also volunteered their own children for the experiment. In addition, radio announcers interviewed members of Hammon’s team, and journalists attended a clinic demonstration where researchers submitted to injections to show their faith in the study.

Like doctors, Utah residents strongly supported Hammon’s study. Long lines snaked around the clinics, as parents accompanied their children to the injection tables. Although the success of Hammon’s study hinged on accurate clinical records and adherence to the protocol, local enthusiasm began to undermine data quality. Some parents illicitly attended more than one clinic to increase the likelihood of their children receiving gamma globulin over placebo, which undermined the statistical merit of the study. Although Hammon could not determine the number of multiple enrolments, the practice highlighted a tension between scientific accuracy and the hopes of desperate parents. In an effort to prevent these multiple enrolments, Utah doctors were asked to wipe a common laboratory phenol reagent on the buttock of each child as a temporary indicator of participation. Meanwhile, other parents chose to attend clinics outside of their enrolment areas to avoid disappointment if supplies were exhausted. Hammon also discovered that local volunteers had secretly redistributed the test sera to the favour of their own communities. Citizen intervention in the supply chain showed the power that some residents exercised over the study and how their actions undermined the creation of scientific knowledge.

Although Hammon was frustrated by the illicit redistribution of the serum and the multiple clinic enrolments, he remained optimistic about the experiment. More than 5000 children had been injected and it was evident that parents and doctors would support scientific research. As statisticians determined that at least another 50 000 children were needed for a significant result, Hammon expanded his experiment.

Because polio was more prevalent during warmer months, the experiment continued the following summer. When a devastating epidemic struck Texas in July 1952, with hundreds of cases of paralysis, Hammon decided that Houston and surrounding Harris County would be an ideal test site.^10^ With foundation support and the assistance of local doctors, politicians, religious leaders and journalists, he actively promoted the gamma globulin study. The publicity program was so successful that some parents not only enrolled their children at Hammon’s clinics, but then asked their family doctors to administer injections of the blood fraction afterward. Hammon attempted to discourage this practice, because it threatened the accuracy of his data set. When such efforts failed, he moved his injection clinics out of noncompliant areas. In addition, some Texas pharmaceutical suppliers built on Hammon’s publicity campaign by promoting their own gamma globulin supplies, leading to sales of more than 20 000 mL of the blood fraction to local residents. Of the 35 000 children enrolled in the Texas study, Hammon could not be certain how many had received private doses of gamma globulin. An effective marketing campaign, combined with parental anxiety at a time of crisis, coalesced to shatter the statistical value of the gamma globulin study.

Pressure to obtain more data led Hammon and the foundation to expand the study to Iowa and Nebraska, where they achieved better compliance to the protocol. Although Hammon enrolled an additional 15 000 children at these test sites, it was clear that the experimental data were already tainted. Hammon’s lack of power over trial conduct and compliance, combined with the foundation’s focus on promoting the experiment, created a wave of optimism that was difficult to contain. Hammon was forced to assess data of dubious quality. When the results of the experiment were finally published, Hammon’s decision not to acknowledge these serious shortcomings facilitated a claim that gamma globulin worked for the prevention of polio paralysis under epidemic conditions.^1^–^5^ Because the experiment had been expensive to undertake, other researchers could not corroborate the results. When the foundation trumpeted the scientific success of the study, Hammon became America’s foremost polio warrior.

With the prevailing optimism about gamma globulin, the foundation spent millions of dollars to procure hundreds of gallons of the blood fraction for a national polio immunization program in 1953 and 1954. More than 220 000 children received injections across America, and 1 728 700 mL gamma globulin were administered. Although many parents and health professionals had no reason to doubt the value of the blood fraction to fight polio, evidence began to emerge that it was not effective. When data were collected about the national program in 1953, the World Health Organization Expert Committee on Poliomyelitis and the United States Public Health Service delivered cutting indictments.

The embarrassment surrounding the ill-fated use of gamma globulin was salvaged by the field testing of Dr. Jonas Salk’s killed-virus vaccine. Foundation officials called on their experience with gamma globulin when designing the Salk polio vaccine trial. They were confident that an enormous civilian experiment was possible, because the gamma globulin study had shown that parents would volunteer their children to an experiment that did not guarantee efficacy or safety. They also refined publicity materials, including films, radio announcements and posters. In an effort to increase the perceived scientific rigour and impartiality of the test, foundation officials established the Vaccine Evaluation Center at the University of Michigan under the leadership of Dr. Thomas Francis Jr.

Following a massive trial, Francis declared the Salk vaccine to be safe and effective and it was licensed on Apr. 12, 1955. Unlike gamma globulin, it could be administered before an epidemic and provided durable immunity to all three types of poliovirus. Although the vaccine marked a major milestone in the polio eradication effort, the earlier testing of gamma globulin offered important lessons for researchers and their sponsoring agencies with respect to how marketing and parental anxiety could inadvertently affect the generation of accurate scientific evidence.
